# Immunization attitudes, opinions, and knowledge of healthcare professional students at two Midwestern universities in the United States

**DOI:** 10.1186/s12909-019-1678-8

**Published:** 2019-07-02

**Authors:** Lauren L. Dybsand, Kylie J. Hall, Paul J. Carson

**Affiliations:** 1 Center for Immunization Research and Education, Department of Public Health, North Dakota State University, Dept 2662, PO Box 6050, Fargo, ND 58108-6050 USA; 2 Sanford Health, Fargo, ND 58104 USA

**Keywords:** Healthcare professional education, Student, Vaccination, Attitudes, Knowledge, Vaccine hesitancy

## Abstract

**Background:**

In addition to administering vaccinations, healthcare professionals (HCPs) also play a crucial role in providing education and advocacy to the public regarding immunizations. Yet, many current and future HCPs are unprepared or reluctant to address the vaccine conversation with hesitant patients. Doctors, pharmacists, and nurses are all recognized as the most trusted sources of vaccine information. By comparing future HCPs in these three distinct programs, we can better understand where potential gaps may lie in their training and education. With insight from students, potential changes to curriculum can improve future HCPs ability to address vaccine hesitancy in their respective careers. The objective of this study was to assess and compare the knowledge, attitudes, and opinions of HCP students on the topic of immunization.

**Methods:**

A cross-sectional survey was conducted in 2017 to assess students in nursing, medical, and pharmacy programs at two universities in the state of North Dakota in the United States. The survey assessed six key themes: 1) demographic information; 2) basic vaccine knowledge; 3) vaccine hesitancy; 4) likelihood to recommend vaccines; 5) confidence in addressing vaccine-related topics with patients; 6) an appraisal of the education they have received on vaccinations.

**Results:**

The survey was completed by 223 participants (overall response rate = 23.7%). Results indicated that vaccine-related knowledge varied greatly by program; high knowledge scores were achieved by 74.3% of medical students, 62.7% of pharmacy students, 57.1% of doctor of nursing practice (DNP) students, and 24.7% of bachelor of science in nursing (BSN) students. Over a third (34.2%) of BSN students believed that the current recommended immunization schedule places undue burden on a child’s immune system, versus only 4.3% of medical students. Additionally, 54.2% of participants believed that spreading out recommended vaccines over several visits was an appropriate means of reducing parental stress about vaccinating.

**Conclusions:**

Participant responses suggest that negative attitudes, lack of knowledge, and general discomfort exist across all programs, but especially among nursing students, regarding vaccination. Our findings indicate potential areas where targeted interventions could be implemented to better equip future HCPs in their ability to discuss and educate the public regarding vaccination.

**Trial registration:**

#PH17173

**Electronic supplementary material:**

The online version of this article (10.1186/s12909-019-1678-8) contains supplementary material, which is available to authorized users.

## Background

Immunization has resulted in a dramatic decline in morbidity and mortality caused by infectious disease [1, 2]. Yet, there is an increasing rate of vaccine hesitancy in the United States (U.S.), with more parents requesting delayed immunization schedules or withholding from vaccinating their children altogether [1, 3, 4]. Additionally, vaccine hesitancy is not only present in the general population, but present in healthcare professionals (HCPs) and has been shown to influence their vaccine-related behavior and decisions [5–15].

In the U.S., recent HCP graduates have shown to, in contrast to their older counterparts, have decreased odds of believing that the recommended childhood vaccines were safe and efficacious [8]. Additionally, many practicing providers lack confidence in pre-licensure vaccine safety studies [15]. HCPs that are hesitant and distrust the efficacy, safety or importance of vaccines are unlikely to properly address vaccine concerns of their patients. This can exacerbate and increase doubt among vaccine-hesitant patients and caregivers [12]. These findings are particularly alarming as HCPs are considered to be the most significant influence and the primary source of information for patients and caregivers regarding vaccines [1, 5, 9, 16–19]. Patient trust in their HCP is often associated with their ultimate decision of whether to accept or decline vaccines [4, 9, 18].

Despite the general public’s trust in HCPs’ recommendation for vaccination, studies have shown that many HCPs are unable or reluctant to effectively communicate about vaccine concerns with vaccine-hesitant parents [3, 12, 18–20]. Furthermore, providers have reported feeling dissatisfied when communication with vaccine-hesitant parents comes to an impasse, and view these consultations as challenging and even conflicting with their professional identities [20].

Internationally, various studies have been undertaken to assess immunization knowledge and attitudes of future HCPs [10, 18, 21, 22]. Research has shown that vaccine related knowledge varies substantially by HCP discipline, with a wide variability in immunization curriculum [10, 18, 21, 22]. Findings suggest that students across all disciplines receive inadequate education regarding immunizations and are uncomfortable discussing vaccine side effects with patients [18]. Further, studies have indicated that vaccine-related knowledge gaps and negative attitudes are a continual challenge for future HCPs [10, 18, 21, 22].

While several studies have been conducted in other countries, very little is known on the knowledge, attitudes, and beliefs of HCP students in the U.S. [10, 18, 22]. What studies have been completed have shown that HCP students lack vaccine knowledge and are uncomfortable with counseling patients on vaccination [13, 14]. It is of ever-increasing importance that future HCPs are knowledgeable on the topic of vaccination and are well-equipped to address the increasing rise of vaccine-hesitant beliefs [1, 5, 9, 12, 14, 15, 18, 21–25]. Further, research in the U.S. has focused on specific disciplines (e.g. medical students) and/or specific vaccinations (e.g. human papillomavirus [HPV] vaccine, influenza vaccine) [8, 13, 14, 25, 26]. Comparisons have not been sought between HCP programs [8, 13, 14, 25, 26]. Greater understanding of the training HCPs receive could provide insight into how their vaccine knowledge, attitudes, and beliefs are formed and how to better equip HCP students to address immunizations in their future careers.

The objective of the study was to assess the vaccination attitudes, knowledge, and beliefs of students in HCP programs at two universities in the U.S. Additionally, comparison was sought between disciplines (e.g. medicine, pharmacy, and nursing).

## Methods

In March of 2017, an anonymous, online, cross-sectional survey was sent to students from three HCP programs at two universities in the state of North Dakota in the U.S. The two schools surveyed represent the only graduate-level healthcare professional programs within the state. The first university’s HCP programs include a school of pharmacy and school of nursing, and the second includes a school of medicine. The survey was distributed via email to participants by two administrative faculty from each university on behalf of the researchers. Participants were required to read a consent statement and provide written agreement to participate prior to beginning the survey. Confidentiality was assured through the consent statement. The survey was sent out once to each department without a reminder email. Participation in the study was voluntary but incentivized with the potential to win a gift card. The North Dakota State University Institutional Review Board approved this project.

Survey questions were developed using previous research in the field as a template [13, 18, 22]. Questions were based on validated templates, some changes and additional questions were added by authors to properly assess the themes noted below. A test survey was sent to other experts in the field to assess questions prior to sending survey to participants. Authors made the following changes to the survey, as suggested by the experts: clarified language used in questions, analyzed means in which surveys were scored, and reanalyzed how questions were organized. The survey included a link to a 35–36 question survey, dependent on participant response (Additional file 1). Questions were grouped into six key themes: 1) demographic information; 2) basic vaccine knowledge; 3) vaccine hesitancy; 4) likelihood to recommend vaccines; 5) confidence in addressing vaccine-related topics with patients; 6) an appraisal of the education they have received on vaccinations.

Question response was scored in which high scores represented high vaccine knowledge, low vaccine hesitancy, high likelihood to recommend vaccination, high confidence in addressing vaccine-related topics with patients, and high confidence in education. Likert scale questions were scored in ranges of 1–5 and 1–10. Knowledge based questions were scored as such: correct = 1, Unsure = 0, and Incorrect = − 1. Additionally, Likert scale items were collapsed to reflect overall agreement (combining scores of somewhat agree and strongly agree).

Responses were grouped according to the six key themes identified. Variables were organized by HCP program, and were further summarized by percentages. Comparisons between programs were made using the Fisher’s Exact Test. This statistical technique was chosen due to the small sample size and its use in similar studies completed on the topic [18, 27–29]. Further regression was not completed due to the sample size. All completed responses per question were analyzed independent of respondents’ completion of the entire survey. All analyses used the Qualtrics© version 3–4.2017 data analysis program and XLSTAT© [30, 31].

## Results

Of the 940 invited to participate 223 participants completed the survey (overall response rate = 23.7%). Twenty-four (10.8%) of the participants failed to complete all of the questions in the survey; questions with reduced responses are noted as such in tables. In the School of Nursing, approximately 21.1% (57/270) of BSN (Bachelor of Science in Nursing – four year program) students, 16.7% (10/60) LPN to BSN students (LPN: Licensed Practical Nurse – 12-18 month program), 68.0% (17/25) RN to BSN students (RN: Registered Nurse – two year program), and 35.0% (14/40) of DNP (Doctor of Nursing Practice) students completed the survey. In the School of Pharmacy, approximately 20.4% (52/255) of Doctor of Pharmacy degree (Pharm.D.) students completed the survey. Lastly, approximately 26.0% (75/290) of medical students responded from the School of Medicine and Health Sciences. The LPN to BSN track, RN to BSN track, and BSN track of the nursing department were collapsed for data analysis. (Table 1).

**Table 1:** Demographic Information

Characteristics	Total (*N* = 223)	%
Age		
18–24 years	130	58.3
25–34 years	87	39.0
35–44 years	5	2.2
45–54 years	1	0.5
55–64 years	0	0
≥ 65 years	0	0
Gender^a^		
Male	65	29.3
Female	157	70.7
Health Professional Program^b^		
Medicine	75	33.2
Pharmacy	52	23.8
Pre-Licensure Bachelor of Science in Nursing (BSN) Track	57	25.2
Licensed Practical Nurse (LPN) - Bachelor of Science in Nursing (BSN) Track	10	4.4
Registered Nurse (RN) to Bachelor of Science in Nursing (BSN) Track	17	8.0
Bachelor of Science in Nursing (BSN) to Doctor of Nursing Practice (DNP)	14	6.2

^a^Gender *N* = 222

^b^Two respondents recorded involvement in more than one health professional program

High scores reflected greater confidence in the education received by students on vaccine-related items. The highest score achieved on items associated with assessing education was 25.00 (out of 25.00), the lowest was a 5.00 (out of 25.00). The mean score of all respondents was 21.00. While a majority of responses associated with assessing education were favorable, there was variability on select items by HCP program.

There was a difference in response by participants on the education received on how vaccines work (*p* = 0.046). Education on vaccine testing and approval processes had the lowest reported positive response of all items across all HCP programs. Of note, only 65.3% of medical students, 64.3% DNP students and 56.6% of BSN students somewhat or strongly agreed their program provided adequate training on vaccine testing and approval process. (Table 2).

**Table 2:** Attitude and Beliefs about Vaccines by Healthcare Professional Program (N = 223)

	BSN positive responses^a^ *n* = 84 *n* (%)	DNP positive responses^a^ *n* = 14 *n* (%)	Medicine positive responses^a^ *n* = 75 *n* (%)	Pharmacy positive responses^a^ *n* = 52 *n* (%)	Fisher’s exact test *p* – value^**b**^
Assessing Education					
Program includes adequate training and/or education in:					
Vaccine preventable diseases	76 (90.5)	14 (100)	73 (97.3)	51 (98.1)	0.504
How vaccines work	67 (79.8)	13 (92.9)	72 (96.0)	49 (94.2)	0.046*
The safety of vaccines^**c**^	68 (82.9)	14 (100)	68 (91.9)	50 (96.2)	0.203
Vaccine testing and approval process^**d**^	47 (56.6)	9 (64.3)	49 (65.3)	39 (75.0)	0.418
How to communicate with vaccine-hesitant caregivers	60 (71.4)	13 (92.9)	55 (73.3)	40 (76.9)	0.646
Measuring Hesitancy					
Routine childhood vaccines are safe^c^	77 (92.8)	14 (100)	72 (97.3)	50 (98.0)	0.524
Protective benefits obtained from vaccinating outweigh the risks that may occur as a result of vaccinating^d^	78 (95.1)	14 (100)	72 (97.3)	50 (98.0)	0.973
Vaccines are effective way to prevent many different diseases^c^	80 (96.4)	14 (100)	72 (97.3)	50 (98.0)	0.722
The current # of recommended childhood vaccines places an undue burden on a child’s immune system^e^	26 (34.2)	3 (23.1)	3 (4.3)	4 (8.5)	< 0.0001*
Measuring Likelihood to Recommend Vaccines					
Caregivers should have influence over what vaccines are given to their children^d^	49 (59.0)	5 (35.7)	26 (35.1)	20 (39.2)	0.028*
Spreading out recommended vaccines over several visits is an acceptable approach to reducing parental stress about vaccinating^d^	45 (54.2)	9 (64.3)	35 (47.3)	23 (45.1)	0.856
State/local vaccination requirements for school/daycare entry are important tools for reducing vaccine preventable diseases in the community^d^	78 (94.0)	14 (100)	70 (94.6)	50 (98.0)	0.888
Caregivers should have the right to request non-medical exemptions from state/local vaccination requirements for school entry^e^	27 (32.9)	2 (14.3)	17 (23.0)	6 (11.8)	0.022*
As a HCP, I believe that I am responsible for advocating the benefit of vaccines and educating patients on the diseases they prevent^e^	78 (95.1)	14 (100)	72 (97.3)	51 (100)	0.878
As a HCP, I believe that my strong recommendation for a vaccination will impact a patient’s decision on whether or not to vaccinate^e^	73 (89.0)	13 (92.9)	62 (83.8)	50 (98.0)	0.148
Getting my annual influenza vaccine is important to me^d^	55 (66.3)	13 (92.9)	64 (86.5)	37 (72.5)	0.062
It is important to engage/encourage HCP to be immunized annually against influenza^d^	64 (77.1)	13 (92.9)	66 (89.2)	42 (82.4)	0.511

^a^Positive responses: Somewhat Agree and Strongly Agree Likert scale responses were collapsed

^b^Fisher exact test was used for analysis with an alpha = 0.05

*p < 0.05

^c^BSN *n* = 82

^d^BSN *n* = 83

^e^BSN *n* = 76, DNP *n* = 13, Medicine *n* = 70, Pharmacy *n* = 47

Four Likert scale statements measured respondents’ vaccine hesitancy. High scores reflected low vaccine hesitancy. The maximum score achieved was 20 (out of 20). The minimum score achieved was 4.00 (out of 20). The mean score of all respondents was 18.66. Although many of the respondents appeared to have low vaccine hesitancy, there was variation in scores between HCP programs on whether or not the current number of recommended vaccines in the childhood schedule places an undue burden on a child’s immune system (*p* < 0.0001). (Table 2) Over a third (34.2%) of BSN students agreed that the current recommended schedule places undue burden on a child’s immune system, compared to 4.3% of medical students.

Eight Likert scale statements measured respondents’ likelihood to recommend vaccines. High scores reflected greater likelihood to recommend vaccines. The maximum score achieved was 40 (out of 40). The minimum score achieved was a 10.00 (out of 40). The mean score of all respondents was 31.93. Overall, most participants scored high on this section, corresponding to a high likelihood to recommend vaccines. Variation in response to whether caregivers should have influence over what vaccines are given to their children (*p* = 0.028) and ability to request non-medical exemptions (*p* = 0.022) was noted between HCP programs. (Table 2).

A difference was also seen by program regarding the importance of receiving an annual influenza vaccine. Nearly all DNP (92.9%) students felt it was important they received an annual influenza vaccine versus only 66.3% of BSN students. Of note, survey responses from almost all programs indicated that respondents were more likely to encourage other HCPs to be immunized annually against influenza than receive the vaccine themselves. For example, there was a difference of 10.8% for BSN students in importance of the individual receiving an influenza vaccination versus importance to engage others to be vaccinated against the virus. (Table 2).

Nearly half of all participants agreed that spreading out recommended vaccines over several visits was an acceptable approach to reducing parental stress about vaccinating. Yet, overall, participants felt they were responsible for advocating the benefits of vaccines, educating patients on the diseases vaccines prevent, and that a strong HCP recommendation could impact a patient’s decision whether or not to vaccinate. (Table 2).

**Fig. 1:**
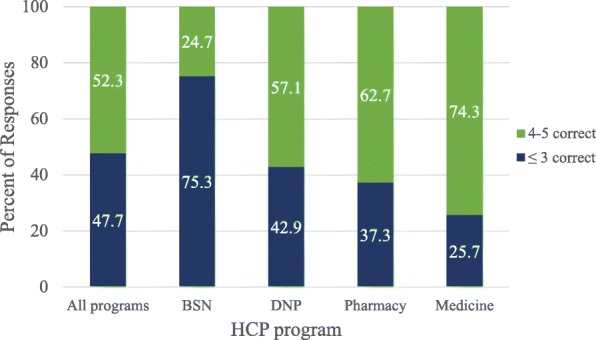
Percent of HCP students that correctly answered knowledge-based questions

There was substantial variation between programs on immunization knowledge. High scores reflected greater vaccine-related knowledge. The maximum score achieved among the five knowledge based questions was a score of 5 (out of 5). The minimum score achieved by a participant was a − 2 (out of 5). The mean score of all respondents was 3.34. Knowledge scores varied significantly by programs (*p* < 0.0001). Medical students scored the highest, with 74.3% answering four to five questions correctly. BSN students rated the lowest, with 24.7% answering four to five questions correctly. (Fig. 1).

Difference in scores was noted between HCP programs on whether to vaccinate or not when a patient presents with mild illness (*p* < 0.0001). Only 32.5% of BSN students answered the question correctly, followed by 68.6% of pharmacy, 78.6% of DNP, and 82.4% of medical programs. Respondents’ ability to properly distinguish the difference in active versus passive immunity varied by program; only 57.1% of DNP students correctly answered the question, compared to 93.2% of medical students (*p* < 0.0001). (Table 3).

**Table 3:** Responses to selected knowledge questions including comparison by program (*N* = 222)

	All Programs (%) *N* = 222 *n* (%)	BSN (%) *n* = 83 *n* (%)	DNP (%) *n* = 14 *n* (%)	Medicine (%) *n* = 74 *n* (%)	Pharmacy (%) *n* = 51 *n* (%)	Association with program (*p*-value)^a^
Hierarchy of evidence question, do the researchers’ studies suggest:^b c^						
Correlation between MMR and autism	2 (0.9)	1 (1.2)	1 (7.1)	0 (0)	0 (0)	
† No link between MMR and autism	173 (78.6)	53 (64.6)	11 (78.6)	65 (89.0)	44 (86.3)	0.001*
Can’t draw a conclusion	45 (20.5)	28 (34.2)	2 (14.3)	8 (11.0)	7 (13.7)	
Patients presenting with mild illnesses, such as cold or bronchitis, should not receive their routine vaccinations.						
True	50 (22.5)	36 (43.4)	3 (21.4)	6 (8.1)	5 (9.8)	
† False	134 (60.4)	27 (32.5)	11 (78.6)	61 (82.4)	35 (68.6)	< 0.0001*
Unsure	38 (17.1)	20 (24.1)	0 (0)	7 (9.5)	11 (21.6)	
Current scientific evidence supports associations between vaccines and chronic conditions such as autism and multiple sclerosis.^d^						
True	1 (0.4)	1 (1.4)	0 (0)	0 (0)	0 (0)	
**†** False	209 (99.5)	72 (98.6)	14 (100.0)	72 (100.0)	51 (100.0)	1.000
Unsure	0 (0)	0 (0)	0 (0)	0 (0)	0 (0)	
Vaccines interact with the immune system and often produce an immune response similar to that produced by the natural infection, but they do not subject the recipient to the disease and its potential complications.						
**†** True	192 (86.5)	67 (80.7)	13 (92.8)	67 (90.5)	45 (88.2)	
False	22 (1.0)	9 (10.8)	0 (0)	7 (9.5)	6 (11.8)	0.046*
Unsure	8 (3.6)	7 (8.5)	1 (7.1)	0 (0)	0 (0)	
The difference in immunity from breastfeeding and immunity from vaccination is that vaccines provide short-term immunologic memory, whereas breastfeeding provides long-term immunologic memory.						
True	16 (7.2)	8 (9.6)	4 (28.6)	2 (2.7)	2 (3.9)	
**†** False	177 (79.7)	55 (66.3)	8 (57.1)	69 (93.2)	45 (88.2)	< 0.0001*
Unsure	29 (13.1)	20 (24.1)	2 (14.3)	3 (4.1)	4 (7.8)	

Correct responses are indicated by an †

^a^Fisher exact test was used for analysis with an alpha = 0.05

*p < 0.05

^b^For full detail on this question, please refer to Additional file 1

^c^BSN *n* = 82, Medicine *n* = 73

^d^BSN *n* = 73, Medicine *n* = 72

Eight questions assessed participants’ confidence in their ability to address a variety of vaccine-related topics with patients, higher scores represented greater confidence. The maximum score achieved by participants was an 80.00 (out of 80.00) and minimum score achieved was an 8.00 (80.00). The mean score for all respondents for all eight questions was 61.80.

There was significant variation by program on respondents’ confidence to: establish ongoing dialogue about vaccines with a patient (*p* = 0.001), discuss patients’ concerns about the effectiveness of vaccines (*p* = 0.004), discuss patients’ concerns about vaccines and autism (*p* < 0.0001), discuss whether vaccines overwhelm the immune system (*p* < 0.0001) and discuss the risk of vaccine preventable diseases with a patient (*p* = 0.021). Generally, DNP students, compared to the other programs, were the most confident at discussing the various vaccine-related themes with patients. There were two notable exceptions: medical students were the most confident at discussing the effectiveness of vaccines with patients, and pharmacy students were the most confident discussing whether vaccines overwhelm the immune system with patients compared to the other programs. (Table 4).

**Table 4:** On a scale of one to ten, with one being the least confident and ten being the most confident, student positive response by program

	All Program positive response^a^ N = 222*n* (%)	BSN positive responses^a^ *n* = 83 *n* (%)	DNP positive responses^a^ *n* = 14 *n* (%)	Medicine positive responses^a^ *n* = 74 *n* (%)	Pharmacy positive responses^a^ *n* = 51 *n* (%)	Fisher’s exact test *p* – value
Benefit of vaccines	143 (64.4)	47 (56.6)	11 (78.6)	52 (70.3)	33 (64.7)	0.372
Risks of vaccines^b^	99 (44.8)	34 (41.5)	8 (57.1)	33 (44.6)	24 (48.0)	0.865
Est. dialogue w/patients^**c**^	117 (52.9)	34 (41.0)	11 (78.6)	51 (68.9)	21 (42.0)	0.001*
Vaccine safety^d^	119 (53.8)	38 (45.8)	7 (53.8)	43 (58.1)	31 (60.8)	0.224
Effectiveness of vaccines	149 (67.1)	46 (55.4)	9 (64.3)	61 (82.4)	33 (64.7)	0.004*
Vaccines and autism	165 (74.3)	48 (57.8)	13 (92.9)	64 (86.5)	40 (78.4)	< 0.0001*
Whether vaccines overwhelm the immune system	128 (57.7)	33 (39.6)	8 (57.1)	49 (66.2)	38 (74.5)	< 0.0001*
Risk of vaccine preventable diseases^c^	171 (77.4)	57 (68.7)	14 (100)	61 (82.4)	39 (78.0)	0.044*

^a^Positive responses: Responses of 8–10 were collapsed to reflect positive response *p < 0.05

^b^BSN *n* = 82

^c^Pharmacy *n* = 53

^d^DNP *n* = 13

## Discussion

Our study confirms and expands on previous study findings. Similar to previous studies, our results suggest variability between HCP programs on several items [8, 13, 14, 18, 21, 22, 25]. In addition, our findings suggest that future HCPs across a number of fields are unprepared to address challenging vaccine-related discussions with patients and caregivers [8, 13, 14, 18, 21, 22, 25]. Statistically significant differences in respondents’ scores were noted on at least one item in all categories when compared by program. Our survey revealed specific deficits of respondents by program and more general insufficiencies across all programs.

The way in which a provider initiates the vaccine conversation can influence a patient or caregiver’s ultimate decision whether to vaccinate or not [3, 14, 25, 32, 33]. In this study, only 68.9% of medical students responded they were highly confident in establishing an ongoing dialogue about vaccines with a patient. It is important that future providers are not only knowledgeable on vaccine-related topics but confident in their ability to initiate and guide the vaccine conversation. Further, similar to previous findings, our results indicated that participants across all three HCP programs lacked confidence in addressing the risks of vaccines [18]. It is important that HCP students are comfortable with addressing both real and perceived risks associated with vaccination.

Pharmacists also play an essential role in administering and providing information on vaccination [16, 18, 34, 35]. Their knowledge, beliefs, and attitudes can not only influence their own choices but that of the patients and caregivers they encounter [12, 16]. Recently, pharmacies in nearly every state have been approved as alternative vaccine delivery sites in the U.S., and pharmacists are now able to provide a select number of vaccines to adolescents and adults [34]. Studies have shown that one of the most common challenges pharmacists encounter during vaccine administrations is educating patients and caregivers on immunizations. Yet, pharmacists also distinguished patient education and promotion as a fundamental component to a successful vaccine delivery site [34]. The current study had similar findings, with less than half of pharmacy students (42.0%) expressing they were highly confident in establishing an ongoing dialogue about vaccines with a patient.

Registered nurses, like pharmacists, play a key role in educating on and administrating vaccinations [7, 36–39]. Through standing orders, nurses are able to provide patients with vaccinations without the need for an examination or direct supervision from the attending provider at the point of care [39]. Moreover, nurses may set the tone of the vaccine conversation before the provider enters the room. Previous studies have shown that nurses lack knowledge on vaccine-related topics [5, 18, 24, 36, 37, 40–42].

Our results indicated that BSN students scored lower on knowledge-based items compared to other HCP students. Only 24.7% of BSN students answered 4–5 (out of 5) knowledge questions correctly. BSN students’ scores generally suggested higher hesitancy, lower likelihood to recommend vaccines, and lower confidence on vaccine-related topics compared to DNP, medical, and pharmacy students.

In this study, influenza vaccine acquisition was used as a surrogate to determine respondents’ vaccine-related knowledge, attitudes, and hesitancy. Vaccinating HCPs against influenza has been shown to reduce the likelihood of transmitting influenza to their patients, and is universally recommended for all HCPs. Furthermore, HCPs are typically in direct contact with and provide care to individuals that may be at greater risk from the virus [10, 11, 25]. Yet, influenza vaccination rates among HCPs in the U.S. are suboptimal [40, 43, 44]. Our study results indicate significant variation by program on the importance of receiving an influenza vaccine. This is consequential as HCPs who were vaccinated themselves were more likely to recommend vaccination to their patients [7, 45].

Approximately half of respondents across all programs agreed that spreading out the recommended schedule is an appropriate method to reduce parental stress about vaccination. Patient vaccine decisions have been shown to be influenced by HCP recommendations, thus it is important that HCPs are knowledgeable and confident in the recommended schedule. Vaccine refusal or delay not only puts the individual patient at risk for vaccine preventable diseases, but also the wider community [1, 5, 9, 16, 17, 33, 46].

When comparing between HCP programs, our results supported previous findings. Students’ knowledge regarding vaccine-related topics varied significantly [18]. The results predictably indicated that medical students overall answered the most questions correctly, corresponding to the increased education received during their training [18]. Yet, knowledge-based questions in the survey assessed basic principles of immunization, topics that HCP students from all three disciplines should be comfortable with answering. Approximately half of respondents answered 3 or less (out of 5) knowledge-based questions correctly, suggesting a need for increased education related to the topic across all disciplines. DNP students generally had the most positive assessment of the education they receive, were the least hesitant, and were the most likely to recommend vaccines compared to students in the other programs assessed.

Future research could expand the themes addressed and the number of programs assessed. Specifically, questions could be added to explore where students source their information, the curriculum used, parental status, year of training, and their vaccination status. Furthermore, participants from additional HCP programs, fields, universities, and practicing HCPs could provide insight to future research and improve generalizability of findings [11, 23, 25].

The World Health Organization has classified vaccine hesitancy as one of the top ten threats to global health [47]. Further emphasizing the need for future HCPs to be well equipped to confidently and knowledgeably address immunization in their future careers. Research conducted both in the U.S. and internationally have shown that education focused on providing future HCPs with strategies to address vaccine hesitancy, along with education with multidisciplinary teams, can significantly improve students’ knowledge and confidence related to immunizations [25, 26].

This study’s results indicate where potential intervention across all HCP programs could improve students’ ability to address the six themes analyzed in the survey. Potential improvements to HCP curriculum could be made by properly assessing potential gaps in current students education. A majority of the students assessed in this survey, while only from two universities, will stay in North Dakota. Thus, representing a HCP workforce for an entire state. By improving their ability to address vaccine hesitancy, they can have a meaningful impact on assuring high vaccination rates in the state.

This study had several limitations. First, the study had a small sample size from two universities, which may create response bias and limit its generalizability. Consequently, although students were asked, they could not be assessed by their stage of training in their respective programs. Yet, the two schools assessed represent the only schools with graduate level healthcare-related programs in the state of North Dakota. Second, despite the fact that the survey was developed using validated and reliable templates, the slight adjustments and additional questions could influence the validity and reliability of the final survey participants’ completed. However, our findings paralleled that of previous research completed on the topic, thus adding promise to the validity of the tool. Third, the lack of assessment of the curriculum used in the three HCP programs limits the comparison. Further, survey results may have been influenced by response bias. Our team was unable to assess non-respondents; their responses may have varied substantially to those that opted to participate. Lastly, the self-reporting nature of the survey may lend to acquiescence and/or social desirability bias.

## Conclusion

Our results suggest that negative attitudes, lack of knowledge, and general discomfort exist across all programs regarding immunization. In particular, BSN students had suboptimal performance in all six themes assessed. While medical students performed well on knowledge based questions, their responses to questions related to recommending vaccinations indicate where improvements could be made in their training. The DNP students generally performed well on survey themes, but still lacked immunization knowledge. All of these findings indicate that HCP students are a key audience for vaccine-related communication strategies and training.

It is important to assure that all HCPs understand the importance of vaccinating and feel confident recommending vaccines to their patients. With a majority of students assessed representing the future HCP workforce for an entire state, the findings provide meaningful insight into their knowledge, attitudes, and beliefs related to immunization. The analysis undertaken in this survey illuminates where action can be taken to improve training students receive in all three programs assessed. The information HCPs provide, and the way in which they provide it, can dramatically influence their patients’ ultimate vaccine decision. Our findings provide insight on where gaps may exist in various HCP training programs. This information may be useful for improving curriculum and training related to vaccination, and increasing vaccination confidence in future HCPs.

## Additional file

Additional file 1: Survey used in study. (DOCX 21 kb)

## Data Availability

The datasets generated and analyzed during the current study are not publicly available due to participants privacy, but are available from the corresponding author on reasonable request.

## Abbreviations

BSN: Bachelor of Science in Nursing
DNP: Doctor of Nursing Practice
HCP: Healthcare professional
LPN: Licensed Practical Nurse
RN: Registered Nurse

## References

[1] Siddiqui M, Salmon DA, Omer SB (2013). Epidemiology of vaccine hesitancy in the United States. Hum Vaccines Immunother.

[2] Center for Disease Control and Prevention (2011). Ten Great Public Health Achievements - United States, 2001-2010. MMWR Morb Mortal Wkly Rep.

[3] Hough-Telford C, Kimberlin DW, Aban I, Hitchcock WP, Almquist J, Kratz R (2016). Vaccine delays, refusals, and patient dismissals: a survey of pediatricians. Pediatrics..

[4] Committee National Vaccine Advisory (2015). Assessing the State of Vaccine Confidence in the United States: Recommendations from the National Vaccine Advisory Committee. Public Health Reports.

[5] Vorsters A, Tack S, Hendrickx G, Vladimirova N, Bonanni P, Pistol A (2010). A summer school on vaccinology: responding to identified gaps in pre-service immunisation training of future health care workers. Vaccine..

[6] Suryadevara M, Handel A, Bonville CA, Cibula DA, Domachowske JB (2015). Pediatric provider vaccine hesitancy: an under-recognized obstacle to immunizing children. Vaccine..

[7] Paterson P, Meurice F, Stanberry LR, Glismann S, Rosenthal SL, Larson HJ (2016). Vaccine hesitancy and healthcare providers. Vaccine..

[8] Mergler MJ, Omer SB, Pan WKY, Navar-Boggan AM, Orenstein W, Marcuse EK (2013). Association of vaccine-related attitudes and beliefs between parents and health care providers. Vaccine..

[9] Kasting ML, Wilson S, Dixon BE, Downs SM, Kulkarni A, Zimet GD (2016). A qualitative study of healthcare provider awareness and informational needs regarding the nine-valent HPV vaccine. Vaccine..

[10] Jennings AR, Burant CJ (2013). Influenza vaccination knowledge and perceptions among veterans affairs nurses. Am J Infect Control.

[11] Mallari J, Goad J, Wu J, Johnson K, Forman T, Neinstein L (2007). Knowledge, attitudes, and practices regarding influenza vaccination among health professional students. J Am Pharm Assoc.

[12] Dubé E (2017). Addressing vaccine hesitancy: the crucial role of healthcare providers. Clin Microbiol Infect.

[13] Betsch C, Wicker S (2012). E-health use, vaccination knowledge and perception of own risk: drivers of vaccination uptake in medical students. Vaccine..

[14] Afonso NM, Kavanagh MJ, Swanberg SM, Schulte JM, Wunderlich T, Lucia VC (2017). Will they lead by example? Assessment of vaccination rates and attitudes to human papilloma virus in millennial medical students. BMC Public Health.

[15] O’Leary ST, Allison MA, Stokley S, Crane LA, Hurley LP, Kempe A (2013). Physicians’ confidence in vaccine safety studies. Prev Med.

[16] Freed GL, Clark SJ, Butchart AT, Singer DC, Davis MM (2011). Sources and perceived credibility of vaccine-safety information for parents. Pediatrics..

[17] Karafillakis E, Dinca I, Apfel F, Cecconi S, Wűrz A, Takacs J (2016). Vaccine hesitancy among healthcare workers in Europe: a qualitative study. Vaccine..

[18] Pelly LP, Pierrynowski MacDougall DM, Halperin BA, Strang RA, Bowles SK, Baxendale DM (2010). The vaxed project: an assessment of immunization education in Canadian health professional programs. BMC Med Educ.

[19] Jelleyman T, Ure A (2004). Attitudes to immunisation: a survey of health professionals in the Rotorua District. N Z Med J.

[20] Berry NJ, Henry A, Danchin M, Trevena LJ, Willaby HW, Leask J (2017). When parents won’t vaccinate their children : a qualitative investigation of australian primary care providers’ experiences. BMC Pediatr.

[21] Lehmann BA, Ruiter RAC, Wicker S, Chapman G, Kok G (2015). Medical students’ attitude towards influenza vaccination. BMC Infect Dis.

[22] Costantino C, Amodio E, Calamusa G, Vitale F, Mazzucco W. Could university training and a proactive attitude of coworkers be associated with influenza vaccination compliance? A multicentre survey among Italian medical residents. BMC Med Educ. 2016;16(38):1–6. Available from: https://bmcmededuc.biomedcentral.com/articles/10.1186/s12909-016-0558-8. 10.1186/s12909-016-0558-8 PMC4734859 26830337

[23] Ko K, Kim S, Kim S, Son KY, Lee J, Lee DR (2017). Knowledge, current status, and barriers toward healthcare worker vaccination among family medicine resident participants in a web-based survey in Korea. Korean J Fam Med.

[24] Hunt C, Arthur A (2012). Student nurses’ reasons behind the decision to receive or decline influenza vaccine: a cross-sectional survey. Vaccine..

[25] Afonso N, Kavanagh M, Swanberg S (2014). Improvement in attitudes toward influenza vaccination in medical students following an integrated curricular intervention. Vaccine..

[26] Schnaith AM, Evans EM, Vogt C, Tinsay AM, Schmidt TE, Tessier KM (2018). An innovative medical school curriculum to address human papillomavirus vaccine hesitancy. Vaccine.

[27] Gargano LM, Herbert NL, Painter JE, Sales JM, Morfaw C, Rask K (2013). Impact of a physician recommendation and parental immunization attitudes on receipt or intention to receive adolescent vaccines. Hum Vaccines Immunother..

[28] Brewer Noel T., Hall Megan E., Malo Teri L., Gilkey Melissa B., Quinn Beth, Lathren Christine (2016). Announcements Versus Conversations to Improve HPV Vaccination Coverage: A Randomized Trial. Pediatrics.

[29] Opel DJ, Heritage J, Taylor JA, Mangione-Smith R, Salas HS, DeVere V (2013). The architecture of provider-parent vaccine discussions at health supervision visits. Pediatrics..

[30] Qualtrics. Qualtrics. Provo, Utah; 2017. Available from: http://www.qualtrics.com.

[31] Addinsoft SARL. XLSTAT. Paris, FRANCE; 2017.

[32] Opel DJ, Mangione-Smith R, Robinson JD, Heritage J, DeVere V, Salas HS (2015). The influence of provider communication behaviors on parental vaccine acceptance and visit experience. Am J Public Health.

[33] Edwards KM, Hackell JM (2016). Countering vaccine hesitancy. Am Acad Pediatr.

[34] Islam JY, Gruber JF, Lockhart A, Kunwar M, Wilson S, Smith SB (2017). Opportunities and challenges of adolescent and adult vaccination administration within pharmacies in the United States. Biomed Inform Insights.

[35] Strand MA, Davidson KM, Schulze N (2017). Linking pharmacists to the delivery of public health services. J Am Pharm Assoc.

[36] Petousis-Harris H, Goodyear-Smith F, Turner N, Soe B (2005). Family practice nurse views on barriers to immunising children. Vaccine..

[37] Gilca V, Boulianne N, Dube E, Sauvageau C, Ouakki M (2009). Attitudes of nurses toward current and proposed vaccines for public programs: a questionnaire survey. Int J Nurs Stud.

[38] Institute of Medicine. The future of nursing. Washington, DC; 2011. Available from: https://www.ncbi.nlm.nih.gov/pubmed/24983041.

[39] CPSTF. Vaccination Programs: Standing Orders. (Community Guide Recommendation). 2016. Available from: https://www.thecommunityguide.org/findings/vaccination-programs-standing-orders. [cited 2017 Mar 27]

[40] Jaiyeoba Oluwatosin, Villers Margaret, Soper David E., Korte Jeffrey, Salgado Cassandra D. (2014). Association between health care workers’ knowledge of influenza vaccine and vaccine uptake. American Journal of Infection Control.

[41] Rhodes D, Visker J, Cox C, Forsyth E, Woolman K (2017). Public health and school nurses’ perceptions of barriers to HPV vaccination in Missouri. J Community Health Nurs.

[42] Savage C, Kub J (2009). Public health and nursing: a natural partnership. Int J Environ Res Public Health.

[43] Black CL, Yue X, Ball SW, Donahue SMA, Izrael D, de Perio MA (2016). Influenza vaccination coverage among health care personnel — United States, 2015–16 influenza season. MMWR Morb Mortal Wkly Rep.

[44] Santibanez T, Kahn K, Zhai Y, O’Halloran A, Liu L, Bridges C (2016). Flu vaccination coverage United States, 2015–16 influenza season. Center for Disease Control and Prevention.

[45] Zhang J, While AE, Norman IJ (2011). Nurses’ knowledge and risk perception towards seasonal influenza and vaccination and their vaccination behaviours: a cross-sectional survey. Int J Nurs Stud.

[46] Institute of Medicine. The child immunization schedule and safety: stakeholder concern, scientific evidence, and future studies. Washington, D.C: The National Academies Press; 2013. Available from: https://www.ncbi.nlm.nih.gov/pubmed/24901198. 24901198

[47] World Health Organization. Ten threats to global health in 2019. 2019 Jan 14; Available from: https://www.who.int/emergencies/ten-threats-to-global-health-in-2019.

