# Supplementary material for: Immunization attitudes, opinions, and knowledge of healthcare professional students at two Midwestern universities in the United States
Source: BMC Med Educ. 2019 Jul 2;19:242. doi: 10.1186/s12909-019-1678-8 (PMC6604347; doi:10.1186/s12909-019-1678-8)
Supplement: Supplementary file 1 — Survey used in study. (DOCX 21 kb) [file 12909_2019_1678_MOESM1_ESM.docx]

**Appendix A: Survey used in study.**

What is your age (in years)?

- 18 - 24
- 25 - 34
- 35 - 44
- 45 - 54
- 55- 64
- 65 and older

What is your gender?

- Male
- Female

Are you a student or faculty member?

- Student
- Faculty

What health professional program are you a student or faculty member in?

- Medicine
- Pharmacy
- Pre-Licensure Bachelor of Science in Nursing (BSN) Track
- Licensed Practical Nurse (LPN) - Bachelor of Science in Nursing (BSN) track
- Registered Nurse (RN) to Bachelor of Science in Nursing (BSN) Track
- Bachelor of Science in Nursing (BSN) to Doctor of Nursing Practice (DNP)

Display This Question:

If Are you a student or faculty member? = Student

And What health professional program are you a student or faculty member in? = Medicine

What year of medical school are you currently completing?

- Year one
- Year two
- Year three
- Year four
- Year five

Display This Question:

If Are you a student or faculty member? = Student

And What health professional program are you a student or faculty member in? = Pharmacy

What year of pharmacy school are you currently completing?

- P1
- P2
- P3
- P4

Display This Question:

If Are you a student or faculty member? = Student

And What health professional program are you a student or faculty member in? = Pre-Licensure Bachelor of Science in Nursing (BSN) Track

What year of nursing education (excluding the time required to complete prerequisite courses) are you currently completing to receive your BSN?

- Year one
- Year two
- Year three

Display This Question:

If Are you a student or faculty member? = Student

And What health professional program are you a student or faculty member in? = Licensed Practical Nurse (LPN) - Bachelor of Science in Nursing (BSN) track

What semester of the LPN-BSN curriculum are you currently completing?

- Semester one
- Semester two
- Semester three
- Semester four
- Semester five
- Semester six

Display This Question:

If Are you a student or faculty member? = Student

And What health professional program are you a student or faculty member in? = Registered Nurse (RN) to Bachelor of Science in Nursing (BSN) Track

What year of the RN-BSN curriculum are you currently completing?

- Year one
- Year two

Display This Question:

If Are you a student or faculty member? = Student

And What health professional program are you a student or faculty member in? = Bachelor of Science in Nursing (BSN) to Doctor of Nursing Practice (DNP)

What year of the DNP program are you currently completing?

- Year one
- Year two
- Year three

Display This Question:

If Are you a student or faculty member? = Faculty

How many years have you taught in a healthcare-related field?

- Under 5 years
- 5 - 10 years
- 11 - 20 years
- 21 - 30 years
- 31+ years

Display This Question:

If Are you a student or faculty member? = Faculty

Are you responsible for creating, teaching, and/or managing any curriculum that includes content on vaccines, immunizations, or another closely related topic?

- Yes
- No

Display This Question:

If Are you a student or faculty member? = Student

Please state your agreement with each of the statements below:
My professional program (medicine, pharmacy, or nursing) includes adequate training and/or education in the following topic areas:

|  | Strongly disagree | Somewhat disagree | Neither agree nor disagree | Somewhat agree | Strongly agree |
| --- | --- | --- | --- | --- | --- |
| Vaccine preventable diseases |  |  |  |  |  |
| How vaccines work |  |  |  |  |  |
| The safety of vaccines |  |  |  |  |  |
| Vaccine testing and approval process |  |  |  |  |  |
| How to communicate with vaccine-hesitant caregivers/patients |  |  |  |  |  |

Display This Question:

If Are you a student or faculty member? = Faculty

Please state your agreement with each of the statements below:
Students in the professional program of which I am a faculty member (medicine, pharmacy, or nursing) receive adequate training and/or education in the following topic areas:

|  | Strongly disagree | Somewhat disagree | Neither agree nor disagree | Somewhat agree | Strongly agree |
| --- | --- | --- | --- | --- | --- |
| Vaccine preventable diseases |  |  |  |  |  |
| How vaccines work |  |  |  |  |  |
| The safety of vaccines |  |  |  |  |  |
| Vaccine testing and approval process |  |  |  |  |  |
| How to communicate with vaccine-hesitant caregivers/patients |  |  |  |  |  |

For the following statements, please rate your level of agreement.

Routine childhood vaccines are safe.

|  | Strongly disagree | Somewhat disagree | Neither agree nor disagree | Somewhat agree | Strongly agree |
| --- | --- | --- | --- | --- | --- |
| Answer |  |  |  |  |  |

For a majority of the population, the protective benefits obtained from vaccinating outweigh the possible risks that may occur as a result of vaccinating (e.g. side effects, adverse events).

|  | Strongly disagree | Somewhat disagree | Neither agree nor disagree | Somewhat agree | Strongly agree |
| --- | --- | --- | --- | --- | --- |
| Answer |  |  |  |  |  |

Vaccines are an effective way to prevent many different diseases, such as measles, hepatitis B, and polio.

|  | Strongly disagree | Somewhat disagree | Neither agree nor disagree | Somewhat agree | Strongly agree |
| --- | --- | --- | --- | --- | --- |
| Answer |  |  |  |  |  |

The current number of recommended childhood vaccines, when received in accordance with the Advisory Committee on Immunization Practices (ACIP) and the Center for Disease Control and Prevention (CDC) recommended schedule, place an undue burden on a child's immune system.

|  | Strongly disagree | Somewhat disagree | Neither agree nor disagree | Somewhat agree | Strongly agree |
| --- | --- | --- | --- | --- | --- |
| Answer |  |  |  |  |  |

For the following statements, please rate your level of agreement.

Parents and caregivers should have influence over what vaccines are given to their children, even if their opinion and beliefs are counter to scientific evidence currently available regarding vaccination.

|  | Strongly disagree | Somewhat disagree | Neither agree nor disagree | Somewhat agree | Strongly agree |
| --- | --- | --- | --- | --- | --- |
| Answer |  |  |  |  |  |

Spreading out recommended vaccines over several visits versus following the Advisory Committee on Immunization Practices (ACIP) and the Center for Disease Control and Prevention (CDC) recommended vaccine schedule is an acceptable approach to reducing parental stress about vaccinating.

|  | Strongly disagree | Somewhat disagree | Neither agree nor disagree | Somewhat agree | Strongly agree |
| --- | --- | --- | --- | --- | --- |
| Answer |  |  |  |  |  |

State and local vaccination requirements for school and daycare entry are important tools for reducing vaccine preventable diseases in the community.

|  | Strongly disagree | Somewhat disagree | Neither agree nor disagree | Somewhat agree | Strongly agree |
| --- | --- | --- | --- | --- | --- |
| Answer |  |  |  |  |  |

For the following statements, please rate your level of agreement.

Parents and caregivers should have the right to request **non-medical exemptions** (philosophical, moral, and/or religious exemptions) from state and local vaccination requirements for school entry.

|  | Strongly disagree | Somewhat disagree | Neither agree nor disagree | Somewhat agree | Strongly agree |
| --- | --- | --- | --- | --- | --- |
| Answer |  |  |  |  |  |

As a healthcare professional, I believe that I am responsible for **advocating the benefit of** **vaccines** and **educating** patients on the diseases they prevent.

|  | Strongly disagree | Somewhat disagree | Neither agree nor disagree | Somewhat agree | Strongly agree |
| --- | --- | --- | --- | --- | --- |
| Answer |  |  |  |  |  |

As a healthcare professional, I believe that my **strong recommendation** for a vaccination will impact a patient's decision on whether or not to vaccinate.

|  | Strongly disagree | Somewhat disagree | Neither agree nor disagree | Somewhat agree | Strongly agree |
| --- | --- | --- | --- | --- | --- |
| Answer |  |  |  |  |  |

Please rate the following questions based on how important you perceive these actions to be.

Getting my annual influenza vaccine is important to me.

|  | Not at all important | Slightly important | Moderately important | Very important | Extremely important |
| --- | --- | --- | --- | --- | --- |
| Answer |  |  |  |  |  |

It is important to **actively engage** and **encourage** all healthcare workers to be immunized annually with influenza vaccine.

|  | Not at all important | Slightly important | Moderately important | Very important | Extremely important |
| --- | --- | --- | --- | --- | --- |
| Answer |  |  |  |  |  |

On a scale of one to ten, with one being the least confident and ten being the most confident, how would you rate your confidence when:

|  | 1 | 2 | 3 | 4 | 5 | 6 | 7 | 8 | 9 | 10 |
| --- | --- | --- | --- | --- | --- | --- | --- | --- | --- | --- |
| Discussing with a patient the benefits of vaccines |  |  |  |  |  |  |  |  |  |  |
| Discussing with a patient the risks of vaccines |  |  |  |  |  |  |  |  |  |  |
| Establishing ongoing dialogue about vaccines with a patient |  |  |  |  |  |  |  |  |  |  |
| Discussing patient's concerns about the **safety** of vaccines |  |  |  |  |  |  |  |  |  |  |
| Discussing patient's concerns about the **effectiveness** of vaccines |  |  |  |  |  |  |  |  |  |  |
| Discussing patient's concerns about **vaccines and autism** |  |  |  |  |  |  |  |  |  |  |
| Discussing patient's concerns about vaccines and whether or not they **overwhelm the immune system** |  |  |  |  |  |  |  |  |  |  |
| Discussing with a patient the risks of vaccine preventable diseases |  |  |  |  |  |  |  |  |  |  |

A set of investigators hear reports that a group of children developed autism within 6 months of having received the measles, mumps, and rubella (MMR) vaccine.  The researchers investigate and published a case series of 10 children displaying signs of autism within 6 months of receiving the MMR vaccine.  
 
The investigators then completed a second study where they selected 10,000 children in the general public and determined their MMR immunization status.  Children were classified as either vaccinated or unvaccinated.  Then, the investigators identified all children diagnosed with autism in both the vaccinated group and the unvaccinated group.  Finally, statistical analysis was done to compare the prevalence of autism in the vaccinated group compared to the unvaccinated group. In the second study, the investigators found no association between receiving the MMR vaccine and having autism.
 
Considering **both** the first and second study completed by the investigators, which statement most closely reflects your assessment of their findings?

- The investigators' research strongly suggests a correlation between the MMR vaccine and autism.
- The investigators' research strongly suggests no link between the MMR vaccine and autism.
- The investigators' research cannot draw any strong conclusion at this time, and they should consider further studies.

For the following questions, please choose whether or not the statement is true or false. If you are not sure whether the answer is true or false, please select unsure.

Patients presenting with mild illnesses, such as cold or bronchitis, **should not** receive their routine vaccinations.

- True
- False
- Unsure

Current scientific evidence **supports** associations between vaccines and chronic conditions such as autism and multiple sclerosis.

- True
- False
- Unsure

Vaccines interact with the immune system and often produce an immune response similar to that produced by the natural infection, but they do not subject the recipient to the disease and its potential complications.

- True
- False
- Unsure

The difference in immunity from breastfeeding and immunity from vaccination is that vaccines provide short-term immunologic memory, whereas breastfeeding provides long-term immunologic memory.

- True
- False
- Unsure
